# On the Thermochemical Transition Depression of Cellulose Acetate Composite Membranes

**DOI:** 10.3390/polym14163434

**Published:** 2022-08-22

**Authors:** Costas Tsioptsias, George-Romanos P. Foukas, Savvina-Maria Papaioannou, Evangelos Tzimpilis, Ioannis Tsivintzelis

**Affiliations:** Department of Chemical Engineering, Aristotle University of Thessaloniki, University Campus, GR-54124 Thessaloniki, Greece

**Keywords:** cellulose acetate, quercetin, gallic acid, thermochemical transition, hydrogen bonding, electrospinning, thermal behavior

## Abstract

Gallic acid (GA) and quercetin (QU) are two important bioactive molecules with increased biomedical interest. Cellulose acetate (CA) is a polymer derived from cellulose and is used in various applications. In this work, differential scanning calorimetry (DSC), thermogravimetric analysis (TGA) and Fourier transform infrared spectroscopy (FTIR) were used to study the thermal behavior of electrospun CA membranes loaded with quercetin or gallic acid. It was found that gallic acid and quercetin depress the thermochemical transition (simultaneous softening and decomposition) of CA, in a mechanism similar to that of the glass transition depression of amorphous polymers by plasticizers. The extensive hydrogen bonding, besides the well-known effect of constraining polymer’s softening by keeping macromolecules close to each other, has a secondary effect on the thermochemical transition, i.e., it weakens chemical bonds and, inevitably, facilitates decomposition. This second effect of hydrogen bonding can provide an explanation for an unexpected observation of this study: CA membranes loaded with quercetin or gallic acid soften at lower temperatures; however, at the same time, they decompose to a higher extent than pure CA. Besides optimization of CA processing, the fundamental understanding of the thermochemical transition depression could lead to the design of more sustainable processes for biomass recycling and conversion.

## 1. Introduction

Cellulose acetate (CA) is one of the most popular cellulose esters, which in turn are among the most popular derivatives of cellulose. In general, the development of cellulose derivatives aims to surpass the issues of cellulose’s limited processing ability arising from lack of thermal transitions prior to decomposition and poor solubility in a wide range of solvents [1,2]. CA membranes have been used and investigated for a broad range of applications. More traditional applications include the use of CA (and cellulose triacetate) membranes in separation processes, such as water treatment [3] and the production of cigarette filters [4]. More recent applications include gas separation by hollow fiber membranes [5], composite CA-ionic liquid membranes [6] or other composite-CA membranes [7,8,9,10,11,12]. In the last two decades, fibrous electrospun CA membranes have attracted significant attention in biomedical applications [2,13,14,15,16], e.g., as drug carriers and tissue-engineering scaffolds.

Among the substances that are used in such biomedical applications are the pharmaceutical quercetin (QU) and the nutraceutical 3,4,5-trihydroxybenzoic acid (usually mentioned as gallic acid, GA). Suggestively, quercetin was recently loaded in poly (vinyl alcohol) (PVA) elecrtospun fibers [17], and gallic acid was loaded in hydroxypropyl methylcellulose nanofibers [18]. QU is a flavonoid compound that belongs to the flavonols, one of the main classes of the flavonoids, and presents anti-oxidant, anti-inflammatory and antibacterial activity [17]. Similar properties are exhibited by gallic acid [19]. Besides biomedical applications, GA has even been tested as an antioxidant for biodiesel [20]. Both GA and QU have limited solubility in water; namely, at 25 °C, the solubility of GA in water is 0.015 (mass fraction) [21], whereas for quercetin the respective mole fraction is as low as 5.5 × 10^5^ [22].

Recently, an inability for exhibiting neat melting, observed as simultaneous softening and decomposition, was first recognized in cellulose acetate butyrate (CAB), and the term “glass chemical transition” was suggested [23]. Later, this behavior was recognized to occur in CA and PVA, and the broader term “thermochemical transition” was used [24]. In more detail, the endothermic effect detected in the differential scanning calorimetry (DSC) curves of these polymers (CA, CAB and PVA) was erroneously attributed to melting in many literature studies. As shown [23,24], their softening is not neat melting, but a peculiar effect accompanied by a minor extent of decomposition. Further, for CAB and CA, it was shown that the endothermic effect at lower temperatures, around 100 °C, does not primarily arise from water evaporation, but is related to the enthalpy of esterification between free acid molecules and hydroxyl groups of cellulose esters [24]. In the literature, similar peculiarities in the thermal behavior can be detected, e.g., for lithium potassium tartrate monohydrate [25]. The vast majority of substances exhibiting thermochemical transition are substances with increased hydrogen bonding.

It is known that small molecules that interact with polymers [26] act as plasticizers and depress the polymer’s glass transition temperature [27]. The plasticization effect has been reported also for CA, e.g., by the addition of Nickel (II) nitrate [28]. Molecules such as QU and GA (with many hydroxyl groups, OH) can strongly interact with CA through hydrogen bonding and, thus, they might present a plasticization effect. Since recent findings show that CA exhibits neither glass transition, nor melting point, but thermochemical transition [24], it would be interesting to find plasticizers to facilitate the processing of CA at lower temperatures. In addition, the depression of thermochemical transition could allow the recycling of CA and similar biomass-based wastes at lower temperatures and, thus, could contribute to saving energy. Consequently, it would be interesting to study CA electrospun membranes loaded with QU or GA, not only due to their increased biomedical potential, but also for (re)examining the thermal behavior of CA under the recent finding of thermochemical transition.

Thus, the scope of this study was to investigate the thermal behavior of CA composite membranes loaded with gallic acid or quercetin under this new prism. In particular, we examined whether QU and GA can depress the thermochemical transition temperature of CA, and how they affect decomposition during the thermochemical transition. This is the first study reporting the thermal behavior of cellulose acetate composites that considers CA as exhibiting a thermochemical transition (simultaneous softening and decomposition) and not a neat melting, or glass transition.

## 2. Materials and Methods

### 2.1. Materials

Cellulose acetate (CA), 30,000 (GPC) average molecular weight and 39.8 % wt. acetyl content, was obtained from Sigma Aldrich(St. Louis, MI, USA). Quercetin (QU), solid (powder), with purity >95% (HPLC), and gallic acid (GA), assay 97.5, were purchased from Sigma Aldrich(St. Louis, MI, USA). *N,N* dimethyl formamide, with purity >99%, was obtained from Alfa Aesar (Haverhill, MA, USA), and acetone, with purity >99.5% (GC), from Sigma Aldrich (St. Louis, MI, USA). Potassium bromide (KBr) with purity >99.5 wt.% was purchased from Chem-Lab (Bunkyo City, Tokyo). KBr was dried for 3 h at 140 °C prior to use. All other chemicals were used as received. 

### 2.2. Polymer Solutions and Electrospinning

The polymer solutions for the electrospinning process were prepared using a mixture of acetone with *N,N*, dimethyl formamide (2:1 volume ratio) as a solvent. In all cases, the concentration of CA was 20 % *w*/*v*. Appropriate amounts of quercetin were added to the solutions, to obtain loadings of 1.5, 3 and 5 wt.%, based on CA weight. Similarly, appropriate amounts of gallic acid were used in order to obtain 1, 3 and 5 wt.% loadings, based on CA weight. As mentioned in the introduction, quercetin and gallic acid exhibit increased interest for biomedical applications. However, these molecules are very interesting also from a materials science and physicochemical point of view, since they contain a high number of hydroxyls, capable for hydrogen bonding, in their molecules (there are four OH groups in the molecule of gallic acid and five in the molecule of quercetin). Such molecules can strongly interact with the respective groups and other polar groups of CA. Such interactions may have a significant influence on the thermal behavior of CA. For these reasons, gallic acid and quercetin were chosen for the fabrication of the composite membranes. In contrast to microcomposites, in nanocomposites, low additive concentrations are typically used. Further, for biomedical applications, typical loadings are in the range of 1–5 wt.% [16]. Thus, the maximum loading for the CA composite membranes was chosen to be 5 wt.%, and two intermediate contents (1 and 3 wt.%) were also studied.

The experimental setup for the electrospinning experiments, a self-assembled apparatus, consists of a syringe pump for the controlled injection of the polymer solution (Harvard Apparatus, model: 2274), a high-voltage source (Spellman High Voltage DC Supply) and a rotating drum that is used as a collector for the fibers, rotating at about 25 rpm. All experiments were performed using a glass syringe of 10 mL internal volume and a 21G needle (internal diameter equal to 0.51 mm). The polymer solutions were electrospun separately, applying 16.5 kV for about 4 hr. The distance between the nozzle and the collector was 5 cm, and the solution flow rate was 1 mL hr^−1^.

### 2.3. Characterization

The fibrous structures produced were examined using Scanning Electron Microscopy (SEM) (model: JEOL, JSM-840A). All surfaces were coated with carbon. The fiber size distributions were obtained using image analysis through the ImageJ Software (version 1.32j, National Institutes of Health, New York USA. In all cases, fibers from more than three images were analyzed, and distributions were obtained by measuring the diameter of more than 500 fibers. 

Thermal degradation of the fibrous structures was examined using a Shimadzu TGA-50 thermogravimetric analyzer (TGA). Measurements were performed using a heating rate of 10 °C min^−1^ under a constant nitrogen flow of 20 cm^3^ min^−1^. The sample weight was higher than 5 mg in all cases.

Thermal properties of the prepared electrospun fibrous structures were obtained using a Shimadzu DSC-50 Differential Scanning Calorimeter (DSC). DSC measurements were performed using a heating rate of 10 °C min^−1^ under a constant nitrogen flow of 20 cm^3^ min^−1^. 

Fourier transform infrared spectroscopy (FTIR) measurements were conducted with a Biorad FTS-175 spectrometer with a resolution of 2 cm^−1^ and 64 scans in absorption mode. The samples were mixed with KBr at a mass proportion of 1/200 (mass of sample/mass of KBr) and were pelletized in a hydraulic press under pressure (100 Bar). The following samples were examined by FTIR: CA membrane, CA loaded with 5% quercetin composite membrane and raw quercetin. In all samples, baseline correction and normalization on a scale from 0 to 1 was applied. Spectra subtraction was also carried out, and the subtracted spectrum was multiplied by a factor of four for visual aid. Curve-free fitting with Gaussian peaks was performed in a positive peak of the subtracted spectrum.

## 3. Results and Discussion

**Figure 1:**
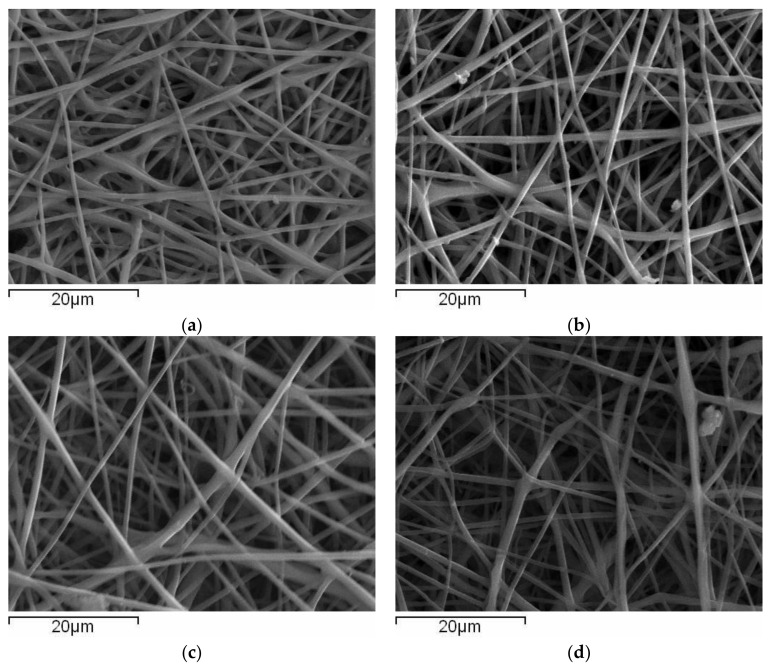
SEM images of electrospun cellulose acetate fibers containing (**a**) 0.0% wt., (**b**) 1.5% wt., (**c**) 3.0% wt. and (**d**) 5.0% wt. quercetin.

**Figure 2:**
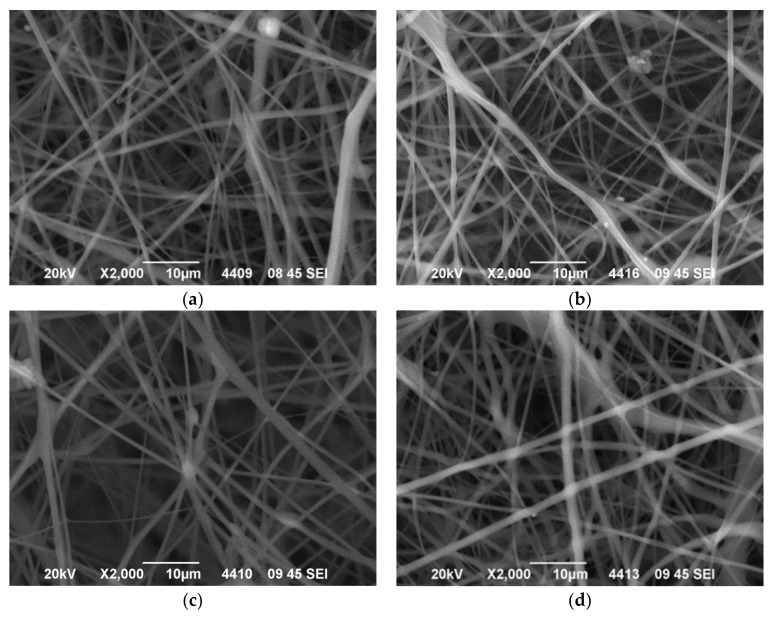
SEM images of electrospun cellulose acetate fibers containing (**a**) 0.0% wt., (**b**) 1.0% wt., (**c**) 3.0% wt. and (**d**) 5.0% wt. gallic acid.

The morphology of the fibrous membranes produced was investigated using Scanning Electron Microscopy (SEM). Representative images regarding the quercetin-loaded fiber mats are provided in Figure 1 for CA-quercetin membranes and Figure 2 for CA-gallic acid membranes. 

Briefly, the fibrous structures obtained did not present bead-on-string defects, but cross-sectionally round fibers and no extensive fiber coalescence. The average diameters of the produced fibers (see Figures S1 and S2 in the Supplementary Information file) did not significantly change with the addition of drugs, most likely due to competitive counterbalanced effects of viscosity increment (which tends to increase fiber diameter) and increase in conductivity (which tends to decrease fiber diameter). In what follows, the thermal behavior of pure substances and composite membranes is examined.

### 3.1. Thermal Behavior of Pure Substances

**Figure 3:**
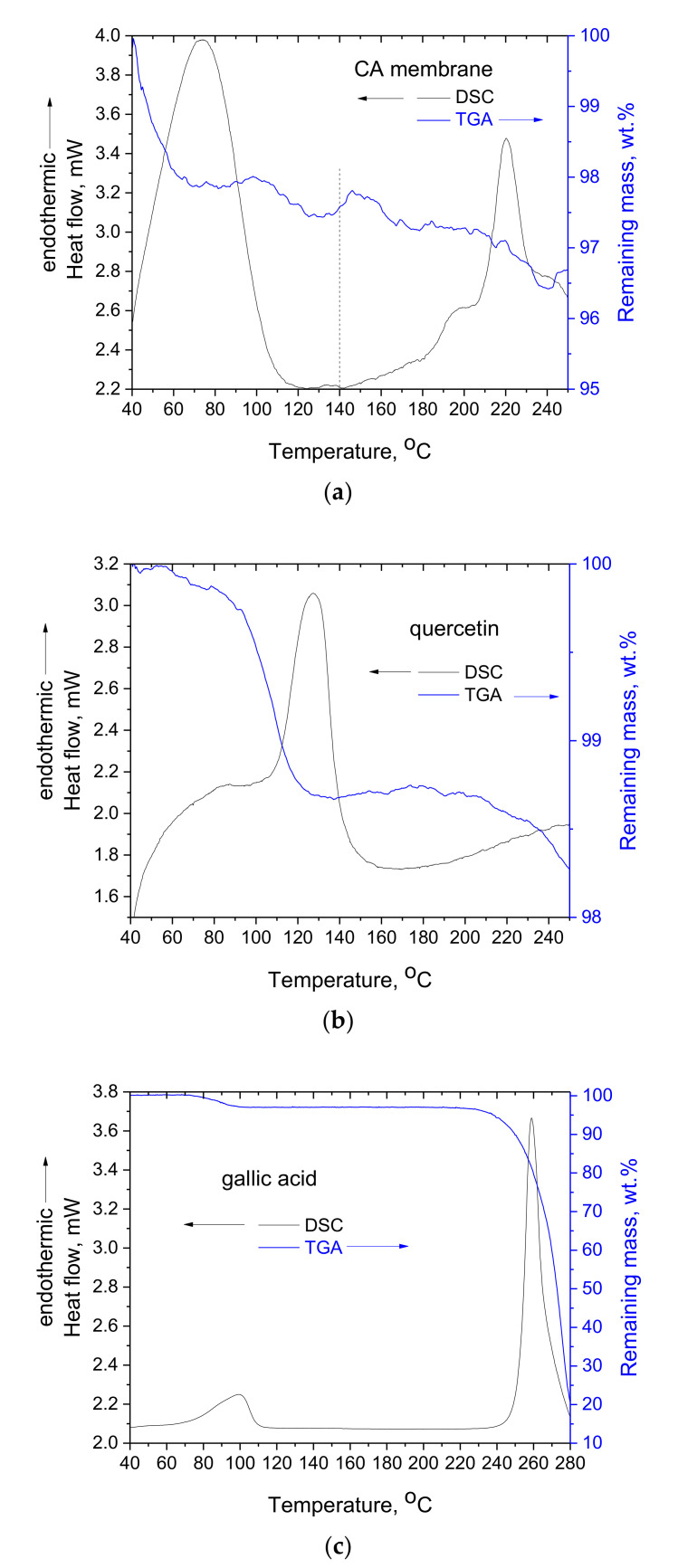
DSC and TGA curves of CA membrane (**a**), quercetin (**b**) and gallic acid (**c**).

In Figure 3, the DSC and TGA curves of raw gallic acid, raw quercetin and pure CA membrane are presented. The thermal behavior of CA was recently thoroughly examined [24]. In both gallic acid and quercetin, the mass loss detected in TGA at around 100 °C and the respective endothermic peak in the DSC plot are related to partial decomposition, and not solely to water removal, according to the literature [20,29]. At higher temperatures, both decompose at around 260 °C for gallic acid and at around 320 °C for quercetin. In both cases, decomposition starts prior to these temperatures being reached. For CA, it was recently reported that the endothermic peak around 100 °C is mainly related to the enthalpy of esterification [24], and at around 220 *°*C, the thermochemical transition occurs and is related to acetyl bond breaking.

Further, for CA it was recently reported [30] that some minor shifting/broadening to higher temperatures of the thermochemical transition occurs when the raw CA powder is dissolved and processed into film. This behavior was attributed to the different conformations and the slight increase in hydrogen bonding that can be achieved during slow re-solidification of CA upon solvent evaporation. On the contrary, the chain conformations in the raw powder are expected to be highly influenced by the conformations of cellulose’s original structure, since CA is typically produced by the heterogeneous esterification of cellulose (cellulose is not dissolved in the organic acid solution) and, thus, a complete disruption of the conformations related to the hydrogen bond network of cellulose is not expected. Thus, the slight increase in hydrogen bonding can explain the shifting of the thermochemical transition temperature towards higher temperatures. Of course, due to the significant degree of substitution in CA samples, there are not enough OH groups to form hydrogen bonds with all the ester group oxygens; thus, a large number of ester groups are free. This can provide an explanation for the fact that before and after re-solidification, only minor differences are observed [30]. In Figure 3a, an opposite behavior is observed (the DSC curve of raw CA powder is not presented since it can be found in various recent publications, e.g., [24,30]). Precisely, a broadening of the DSC peak towards lower temperatures can be observed with an onset temperature around 140 °C. At the same temperature, some mass loss can be observed in the respective TGA curve. Thus, a combined interpretation of DSC and TGA suggests that the simultaneous softening and partial decomposition of CA membranes initiates at lower temperatures than the raw powder sample. Two factors can contribute to this. The first factor is related to the increased surface-to-volume ratio of the electrospun fibrous membranes, which facilitates heat transfer. The second factor can be related to decreased hydrogen bond formation. In contrast to the abovementioned case of film formation upon slow solidification, the solvent evaporation is very rapid (<1 s) in the case of electrospinning. Thus, the CA fibers quickly solidify without the chains having the necessary time to rearrange and form all possible hydrogen bonds. Thus, the CA electrospun membranes exhibit slightly different behavior than the CA raw sample or CA film.

### 3.2. Composite Membranes

#### 3.2.1. Cellulose Acetate-Quercetin Membranes

**Figure 4:**
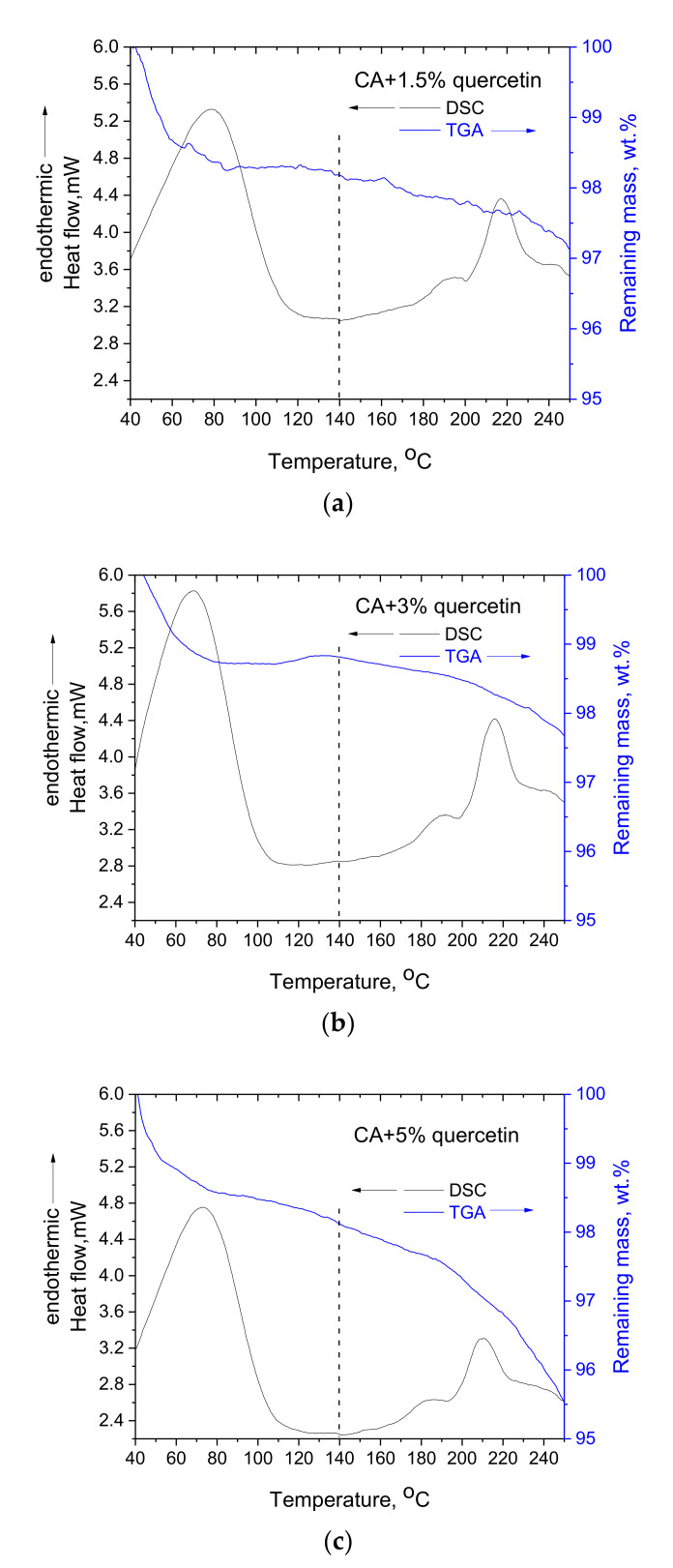
DSC and TGA curves of CA-quercetin composite membranes with quercetin loadings of 1.5 wt.% (**a**), 3 wt.% (**b**) and 5 wt.% (**c**).

In Figure 4, the DSC and TGA curves for the CA-quercetin membranes are presented. The composite membranes exhibit qualitatively the same behavior with the pure CA membrane, i.e., one endothermic peak at around 100 °C, the thermochemical transition at around 220 °C and two respective mass loss stages. In addition, the thermochemical transition in the composite membranes initiates at (approximately) 140 °C, as in pure CA membrane, whereas from the respective TGA curves, it can be seen that this temperature is the onset for the second stage of mass loss. In Figure 4c, for the CA-5% quercetin sample, it can be observed that there is no distinction between the two stages of mass loss, and a continuous decrease in weight occurs. However, it can be also observed that at 140 °C, the mass loss rate increases.

The temperature at the maximum of the thermochemical transition peak, along with the mass loss percentage (wt.%) for pure CA membrane and the three CA-quercetin composite membranes, are presented in Table 1. If the values presented in Table 1 are viewed separately from one another, a clear conclusion is more difficult to be extracted, e.g., for the pure CA membrane and the CA-1.5% quercetin membrane, there is no significant difference in the mass loss observed in the range of 140–250 °C. However, a combined observation of the results presented in Table 1 points out interesting conclusions. First, the thermochemical transition temperature is depressed, and the extent of the depression is positively correlated with the quercetin content. Second, although pure quercetin losses mass around 100 °C, the composite CA-quercetin membranes exhibit lower mass loss than pure CA in the same temperature range (40–100 °C). Third, in the temperature range of 140–250 °C, the composite membranes exhibit higher mass loss. The lower mass loss in the range of 40–100 °C suggests that the addition of quercetin interferes with the esterification reaction. This can occur based on two different contributions: (1) by blocking the availability of CA’s OH through hydrogen bonding or (2) by blocking the free acid groups (formed by hydrolysis).

Regarding the depression of the thermochemical transition and the respective increase in mass loss by increasing the quercetin’s content, (seemingly) contradictive conclusions are derived. Since the thermochemical transition is depressed, the decomposition rate would be expected to be lower for both CA and quercetin due to the lower temperature. Further, the additional mass loss in the composite membranes, compared to the mass loss in the pure CA membrane, cannot be attributed to the addition of quercetin, considering that the latter is just an inert compound. As can be seen in Table 1, in the temperature range of 140–250 °C, quercetin exhibits a mass loss of around 0.5 wt.%. Thus, in the composite CA-5% quercetin membrane, an additional 0.025 wt.% (5 × 0.5/100) mass loss can be justified if quercetin is considered an inert compound. Thus, the respective mass loss of pure CA membrane, which is 1 wt.%, should only increase to 1.25 wt.% for the composite CA-5% quercetin membrane. However, the observed value is quite higher and equal to 2.6 wt.%. Consequently, the behavior of the composite is not just the sum of the behavior of pure substances. This can confirm that the composite membrane presents a single-phase behavior, or, in other words, that CA and quercetin are miscible. Prior to continuing this discussion further, the results for the CA-gallic acid composite membranes are presented.

#### 3.2.2. Cellulose Acetate-Gallic Acid Membranes

**Figure 5:**
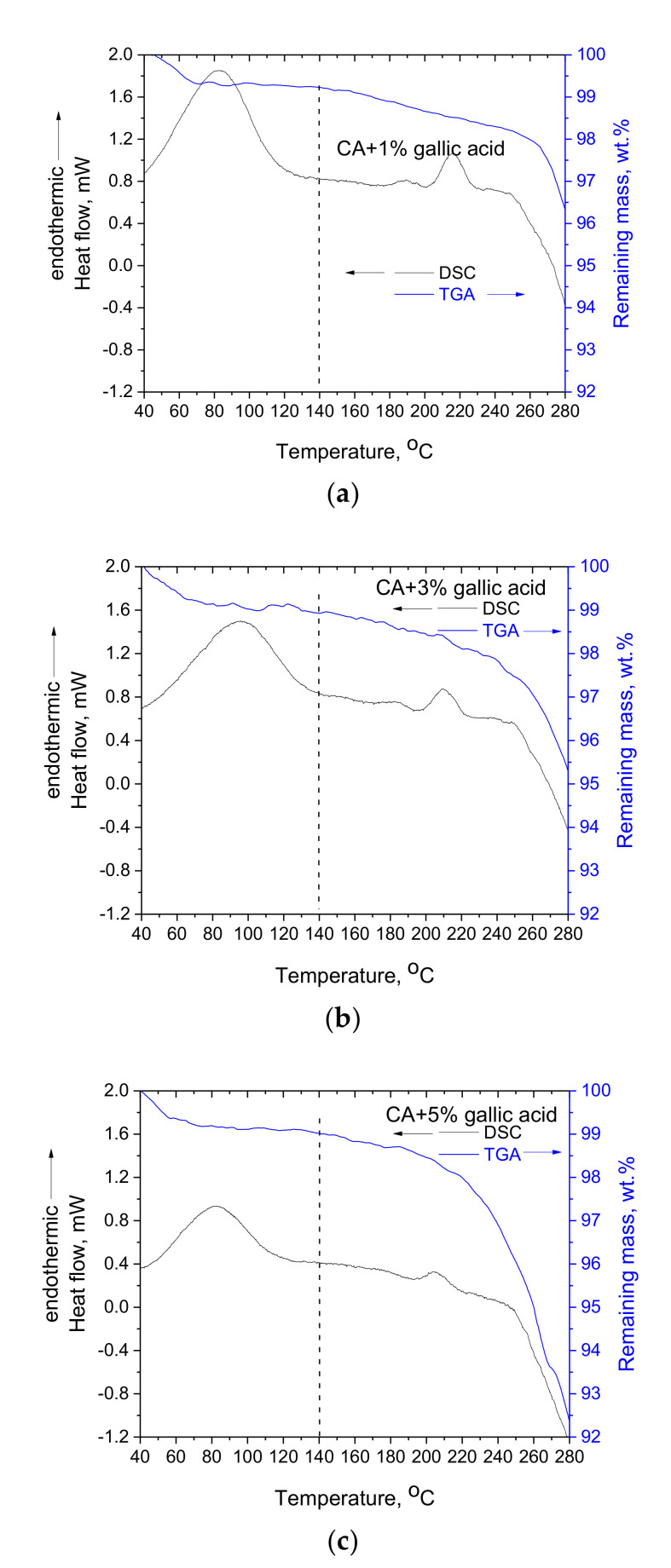
DSC and TGA curves of CA-gallic acid composite membranes with gallic acid loadings of 1 wt.% (**a**), 3 wt.% (**b**) and 5 wt.% (**c**).

The DSC and TGA curves for the three CA-gallic acid composite membranes are presented in Figure 5. The temperature of the maximum of the peak referring to the thermochemical transition, along with the mass loss percentage for pure CA membrane and the three CA-gallic acid composite membranes, are presented in Table 2.

As can be seen in Figure 5 and Table 2 for the CA-gallic acid membranes, similar observations as for the CA-quercetin membranes can be made. Precisely, mass loss initiates to occur around 140 °C, and the thermochemical transition temperature of CA is depressed by increasing gallic acid content. Further, the mass loss at around 100 °C is lower in the composite membranes, whereas the mass loss in the range of 140–250 °C is higher and cannot be attributed simply to the additional mass loss contribution from gallic acid (if this is considered an inert compound). Some small quantitative differences can be observed between gallic acid and quercetin, i.e., in the CA-5% gallic acid membrane, the extent of decomposition and the depression of the thermochemical transition temperature are higher compared to the respective ones of CA-5% quercetin. From these observations, it can be concluded that gallic acid has a stronger “plasticization” capacity for CA than quercetin. This could be attributed to the smaller molecular size of gallic acid. However, qualitatively, the same conclusions are reached, and among the most important ones is the miscibility of CA with gallic acid and quercetin. In what follows, the source of this miscibility is examined, and its effect on the thermochemical transition of CA is discussed.

Before proceeding, it is worth mentioning that in the case of CA-Fe_2_O_3_ composite electrospun membranes, no considerable alteration was reported for the thermal transition of CA [31]. Similarly, cellulose acetate-iron acetate composites were reported to have only slight differences in their thermal transition temperature [10]. On the contrary, for cellulose triacetate-ionic liquid membranes, a tremendous depression of the thermal transition of CA was observed by increasing the ionic liquid content [6]. Ionic liquids present solvating/swelling capacity for CA, since they can strongly interact with its polar groups. The high depression of the thermal transition is related to the high content of ionic liquid (10–50 wt.%). It is worth mentioning that for CA composite membranes, the characterization is typically focused on properties other than the thermal transition, e.g., mechanical properties [9,32], thermal stability [8,9], water contact angle [8,33] and water permeability [34], antimicrobial activity [12], structure investigated by microscopy and XRD [11], etc.

## 4. Further Discussion 

A non-sufficient, but necessary condition for miscibility is the negative Gibbs free energy of mixing (Δ*G_mix_*). The interaction of CA with gallic acid, or quercetin, is expected to occur through hydrogen bonding. In general, in mixtures of unlike molecules, e.g., A and B, a negative enthalpy of mixing (Δ*H_mix_*) is probable if the hydrogen bonding interactions of unlike molecules, that is, the A-B interactions, are stronger than interactions of molecules of the same kind, that is, the A-A and B-B interactions. In the case of CA (with a specific degree of substitution), there are certainly interactions of molecules of the same kind (CA-CA) in terms of hydrogen bonding due to unreacted hydroxyl groups (OH not reacted to form ester groups). However, in the repeating unit of CA, there should be free OH, but also various oxygen atoms, e.g., the two oxygens of the ester group or the oxygen of the glycosidic bond, which could take part in hydrogen bonding with OH groups of other molecules (drug in this case). 

Considering the high degree of acetylation, most of these hydrogen-bonding groups are free (not hydrogen-bonded) in pure CA membranes, whereas some of them form hydrogen bonds with drug molecules upon the addition of quercetin or gallic acid. 

As it was observed and interpreted in a previous study [30], the slow re-solidification of CA after dissolution and, thus, the disruption of the non-equilibrium conformations of CA in raw powder (reminiscent of cellulose structure), lead to an increase in hydrogen bonding in re-solidified CA and cause a small broadening of the thermochemical transition peak towards higher temperatures. Overall, increasing the hydrogen bonding interactions between CA-CA tends to keep chains closer to each other, leading to the elevation of the thermochemical transition temperature.

However, in the case of the gallic acid and quercetin-CA membranes, the increased hydrogen bonding has a different effect (depression instead of elevation of the thermochemical transition temperature) due to the smaller size of the drugs. Gallic acid and quercetin molecules can diffuse through the chains of CA and, as it occurs in the plasticization of amorphous thermoplastic polymers, they increase the polymers’ free volume by increasing the distance of the chains and, thus, enable softening at lower temperatures. 

Differences in the intermolecular interactions can also justify the increased plasticization capacity of gallic acid in comparison with quercetin. Although gallic acid has four OH in each molecule, whereas quercetin has five OH groups, more stereochemical constraints are expected in quercetin due to the significantly larger molecular size, and the smaller molecule of gallic acid is expected to present such constraints to a lesser extent. 

In conclusion, hydrogen bonding between CA-CA tends to keep CA chains close to each other (tending to the elevation of CA’s thermochemical transition temperature), and hydrogen bonding between CA and gallic acid, or quercetin, results in the insertion of smaller molecules between the polymer chains, increasing the distance of CA-CA, causing a depression in CA’s thermochemical transition temperature. 

The remaining question is for what reason the extent of decomposition during thermochemical transition increases, despites the lower temperature. In order to provide an answer to this question, a second effect of hydrogen bonding should be considered. In general, the hydrogen bonding in pure substances has a double effect on the thermochemical transition, i.e., it constrains melting/softening through the strong intermolecular interactions, and it enables/favors decomposition through the weakening of chemical bonds [35]. In the case of a polymer mixture with a low molecular weight substance, as discussed above, in contrast to the pure substance case, softening is enabled due to the plasticization effect of the small molecules. However, the weakening of chemical bonds due to hydrogen bonding is also valid for the case of mixtures. 

**Figure 6:**
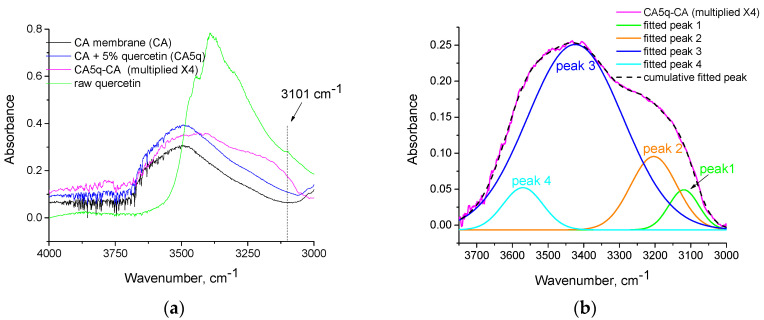
(**a**) FTIR spectra of CA membrane (CA), CA-5% quercetin (CA5q), their subtracted spectrum (CA5q-CA) and raw quercetin in the region 2500–4000 cm^−1^. (**b**) Curve fitting of the positive peak of the subtracted spectrum occurring in the region 3000–3750 cm^−1^.

To study the latter effect, the pure CA membrane, raw quercetin and CA-5% quercetin composite membrane were examined by FTIR. Quercetin was chosen instead of gallic acid, since intense contributions of water are present in the infrared absorption spectrum of gallic acid, complicating the evaluation of the FTIR spectra. For a similar reason, i.e., to facilitate the evaluation, the 5% quercetin was chosen, but it is obvious that the qualitative conclusions derived from the CA-5% quercetin sample can also be valid for the samples of lower quercetin content (or loaded with gallic acid). The FTIR spectra of the CA membrane, the CA-5% composite membrane, along with their subtracted spectrum and the spectrum of raw quercetin, are presented in Figure 6a. The subtracted spectrum has been multiplied by a factor of four to enhance visual evaluation.

As can be seen in Figure 6a, in the O-H stretching region (3000–4000 cm^−1^), a broad positive peak has appeared in the subtracted spectrum. Such a positive peak is expected, since the CA-5% quercetin membrane has increased OH content compared to neat CA, due to the addition of quercetin. However, the maximum absorption for pure quercetin occurs at 3392 cm^−1^ and, although the positive peak of the subtracted spectrum has a contribution from this band, it is obvious that there is a new contribution from OH groups shifted to lower wavenumbers. Curve-free fitting (with Gaussian peaks) was performed in the positive peak of the subtracted spectrum, as illustrated in Figure 6b. The wavenumbers, the areas and the percentage relative areas of the fitted peaks are presented in Table 3.

As can be seen in Table 3 and Figure 6b, at least four peaks are required in order to have a proper fitting of the positive peak of the subtracted spectra. Note that in the fitted peak at 3119 cm^−1^, there is an expected high contribution from = C-H stretching vibrations [36]. However, since the peak is positioned at wavenumbers higher than 3100 cm^−1^, it follows that there is also a contribution from highly shifted hydroxyl groups. Nevertheless, this peak was not taken into account in the normalization of the areas of the peaks. The 78% of the hydroxyl groups in the CA-5% quercetin membrane are positioned at 3423 cm^−1^, that is, very close to the wavenumber of quercetin’s maximum absorption (3392 cm^−1^). However, a countable portion of OH groups (15%) are considerably shifted at lower wavenumbers (3204 cm^−1^). It is known that vibrations of strongly bounded hydroxyls appear at lower wavenumbers than free or less strongly bounded OH groups [36]. The reason for this is that the force constant of the chemical bond is decreased due to the strong physical interactions, meaning that the chemical bond is weakened. This can be readily realized by the vibrational wavenumber ($\tilde{\nu }$) of the molecular oscillator given by the following equation [37]:

(1) $$\tilde{\nu }=\frac{1}{2\pi c}\sqrt{\frac{k}{\mu }}$$

where *c* is speed of light, *k* is the force constant and *μ* is the reduced (effective) mass.

If, in Equation (1), we set the values of the wavenumbers of the abovementioned peaks (with 78% and 15% contributions to the overall positive peak of the subtracted spectrum) and divide the resulting two equations, it follows that:

(2) $$\frac{3570}{3204}=\frac{\frac{1}{2\pi c}\sqrt{\frac{k_{3570}}{\mu }}}{\frac{1}{2\pi c}\sqrt{\frac{k_{3204}}{\mu }}}\Rightarrow \frac{k_{3204}}{k_{3570}}=0.81$$

The force constant is an established approach to express the strength of a chemical bond [38,39]. Thus, from Equation (2), it follows that 15% of the hydroxyl groups in the CA-5% quercetin membrane form chemical bonds that are 19% (1 − 0.81 = 0.19) less strong than the chemical bonds occurring for the majority (78%) of the OH groups. Similarly, the other chemical bonds of groups involved in hydrogen bonding with OH groups are also expected to be weakened to some extent. These weaker chemical bonds are most likely responsible for the increased decomposition during the depressed thermochemical transition of CA in the composite membranes.

From the above, it is suggested that for optimizing the conditions of CA processing, e.g., for decreasing the extent of decomposition during thermochemical transition, plasticizers with small size and low OH content would be preferable. On the other hand, for cellulose acetate waste conversion, e.g., through pyrolysis or gasification, plasticizers with increased hydrogen-bonding capacity will allow for increased decomposition of waste at lower temperatures and, thus, could render the overall process less energy-consuming. 

## 5. Conclusions

The thermal behavior of CA is governed, to a considerable extent, by hydrogen bonding. The fibers, among other reasons, exhibit slightly different behavior from the respective behavior of the raw CA powder, due to the rapid solidification during electrospinning and, thus, to the lack of sufficient time for rearrangement of polymer chains and the formation of the maximum number of hydrogen bonds.

Gallic acid and quercetin exhibit a plasticization capacity for CA membranes. This capacity is higher when gallic acid is used due to its smaller size. Using both plasticizers, extensive hydrogen bonding is expected to occur with various polar groups of CA. s a consequence, the hydrogen boding between these small molecules and CA macromolecules facilitates an increase in the free volume of the polymer matrix, leading to a depression in the thermochemical transition of CA. At the same time, the extensive hydrogen bonding is responsible for the weakening of chemical bonds and, thus, the extent of decomposition is increased despite the lower (depressed) transition temperatures of the composite membranes. Small molecules with lower number of OH than that of gallic acid or quercetin should be explored as potential plasticizers for CA, which will not increase the extent of decomposition during thermochemical transition. Such plasticizers will allow for the processing of CA at lower temperatures with a lower extent of decomposition.

## Figures and Tables

**Table 1:** Thermochemical transition temperature and mass loss within two different temperature ranges for CA and CA-quercetin membranes.

Sample	Thermochemical Transition Temperature, °C	Mass Loss in the Range of 40–100 °C, wt.%	Mass Loss in the Range of 140–250 °C, wt.%
CA membrane	220	2.2	1.0
CA + 1.5% quercetin	217	1.7	1.0
CA + 3% quercetin	216	1.3	1.1
CA + 5% quercetin	210	1.5	2.6
Quercetin	>250	1.3	0.5

**Table 2:** Thermochemical transition temperature and mass loss within two different temperature ranges, for CA and CA-gallic acid membranes.

Sample	Thermochemical Transition Temperature, °C	Mass Loss in the Range of 40–100 °C, wt.%	Mass Loss in the Range of 140–250 *°*C, wt.%
CA membrane	220	2.2	1.0
CA + 1% gallic acid	216	0.7	1.0
CA + 3% gallic acid	210	0.9	1.5
CA + 5% gallic acid	205	0.9	3
Gallic acid	261	3	7.2

**Table 3:** Wavenumber, areas and percentage areas of the fitted peaks presented in Figure 6b.

Fitted Peak	Wavenumber, cm^−1^	Area	% Area
1	3119	5.90	*
2	3204	16.54	15
3	3424	86.34	78
4	3570	8.18	7

* This peak was not included in the normalization, because its main contribution is expected to arise from =C-H vibrations and not O-H vibrations.

## Data Availability

Not applicable.

## Supplementary Materials

The following supporting information can be downloaded at: https://www.mdpi.com/article/10.3390/polym14163434/s1, Figure S1: Average fiber diameters for membranes containing 0.0, 1.5, 3.0 and 5.0 %wt. quercetin; Figure S2: Average fiber diameters for membranes containing 0.0, 1.0, 3.0 and 5.0 %wt. gallic acid.
