# Supplementary material for: On the Thermochemical Transition Depression of Cellulose Acetate Composite Membranes
Source: Polymers (Basel). 2022 Aug 22;14(16):3434. doi: 10.3390/polym14163434 (PMC9416459; doi:10.3390/polym14163434)
Supplement: Supplementary file 1 [file polymers-14-03434-s001.zip › polymers-1871132-supplementary.pdf]

Supporting information for

On the thermochemical transition depression of cellulose acetate composite membranes

Costas Tsioptsias[1], George-Romanos P. Foukas[1], Savvina-Maria Papaioannou[1], Evangelos Tzimpilis[1] and Ioannis Tsivintzelis[1]*

[1] Department of Chemical Engineering, Aristotle University of Thessaloniki, University Campus, GR-54124 Thessaloniki, Central Macedonia, Greece; ktsiopts@gmail.com (C.T.); grfoukas@gmail.com (G.-R. F.); savina94@gmail.com (S.-M. P.); tzimpi@auth.gr (E.T.)

* Correspondence: tioannis@cheng.auth.gr (I.T.)

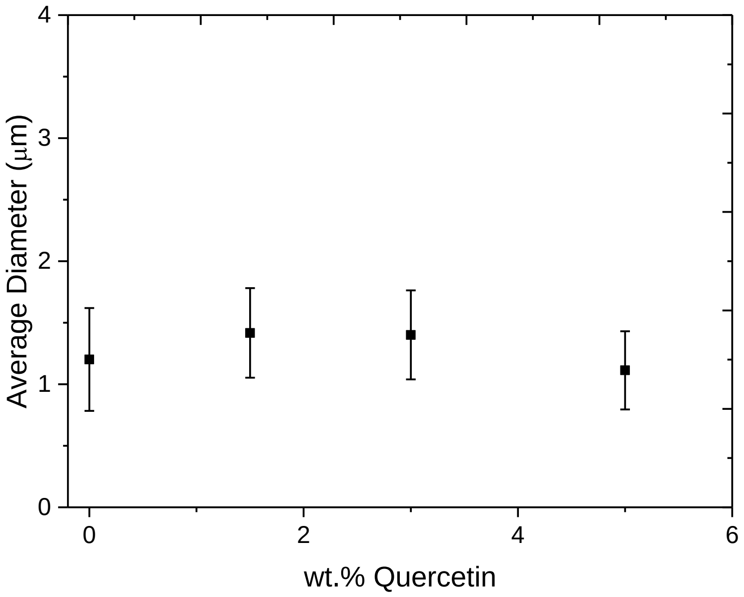

Figure S1. Average fiber diameters for membranes containing 0.0, 1.5, 3.0 and 5.0 %wt. quercetin.

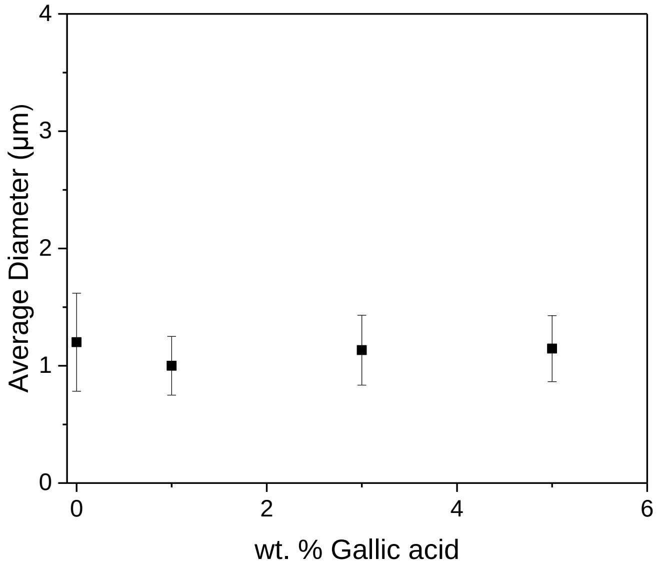

Figure S2. Average fiber diameters for membranes containing 0.0, 1, 3.0 and 5.0 %wt. gallic acid.
